# Cost-Effectiveness of Early Infant HIV Diagnosis of HIV-Exposed Infants and Immediate Antiretroviral Therapy in HIV-Infected Children under 24 Months in Thailand

**DOI:** 10.1371/journal.pone.0091004

**Published:** 2014-03-14

**Authors:** Intira Jeannie Collins, John Cairns, Nicole Ngo-Giang-Huong, Wasna Sirirungsi, Pranee Leechanachai, Sophie Le Coeur, Tanawan Samleerat, Nareerat Kamonpakorn, Jutarat Mekmullica, Gonzague Jourdain, Marc Lallemant, for the Programme for HIV Prevention and Treatment (PHPT) Study Team

**Affiliations:** 1 Faculty of Epidemiology and Population Health, London School of Hygiene & Tropical Medicine, London, United Kingdom; 2 Institut de Recherche pour le Développement (IRD)-Programs for HIV Prevention and Treatment (PHPT), Chiang Mai, Thailand; 3 Department of Immunology and Infectious Diseases, Harvard School of Public Health, Boston, Massachusetts, United States of America; 4 Faculty of Public Health and Policy, London School of Hygiene & Tropical Medicine, London, United Kingdom; 5 Department of Medical Technology, Faculty of Associated Medical Sciences, Chiang Mai University, Chiang Mai, Thailand; 6 Unité Mixte de Recherche 196 Centre Français de la Population et du Développement (INED-IRD-Paris V University), Paris, France; 7 Paediatrics Department, Somdej Prapinklao Hospital, Bangkok, Thailand; 8 Paediatrics Department, Bhumibol Adulyadej Hospital, Bangkok, Thailand; University of Pittsburgh, United States of America

## Abstract

**Background:**

HIV-infected infants have high risk of death in the first two years of life if untreated. WHO guidelines recommend early infant HIV diagnosis (EID) of all HIV-exposed infants and immediate antiretroviral therapy (ART) in HIV-infected children under 24-months. We assessed the cost-effectiveness of this strategy in HIV-exposed non-breastfed children in Thailand.

**Methods:**

A decision analytic model of HIV diagnosis and disease progression compared: EID using DNA PCR with immediate ART (Early-Early); or EID with deferred ART based on immune/clinical criteria (Early-Late); vs. clinical/serology based diagnosis and deferred ART (Reference). The model was populated with survival and cost data from a Thai observational cohort and the literature. Incremental cost-effectiveness ratio per life-year gained (LYG) was compared against the Reference strategy. Costs and outcomes were discounted at 3%.

**Results:**

Mean discounted life expectancy of HIV-infected children increased from 13.3 years in the Reference strategy to 14.3 in the Early-Late and 17.8 years in Early-Early strategies. The mean discounted lifetime cost was $17,335, $22,583 and $29,108, respectively. The cost-effectiveness ratio of Early-Late and Early-Early strategies was $5,149 and $2,615 per LYG, respectively as compared to the Reference strategy. The Early-Early strategy was most cost-effective at approximately half the domestic product per capita per LYG ($4,420 in Thailand 2011). The results were robust in deterministic and probabilistic sensitivity analyses including varying perinatal transmission rates.

**Conclusion:**

In Thailand, EID and immediate ART would lead to major survival benefits and is cost- effective. These findings strongly support the adoption of WHO recommendations as routine care.

## Introduction

In 2011, there were an estimated 330,000 infants newly infected with HIV through mother-to-child transmission (MTCT), over 90% of whom were in sub-Saharan Africa and Asia [1]. Without antiretroviral therapy (ART), up to 50% will die by two years of age, in resource limited settings [2], [3]. The scale up of ART has dramatically reduced HIV-related mortality in children [4]–[6]. However, the risk of early mortality on ART remains high among infants initiating therapy *after* presenting with symptoms or immunosuppression, with 14% to 27% deaths during the first year of therapy [7]–[9]. The landmark CHER trial in South Africa, which randomized asymptomatic HIV-infected infants with CD4>25% (at median of 7 weeks old) to immediate or deferred ART based on WHO 2006 clinical and immune criteria, reported a 76% reduction in mortality and 75% reduction in disease progression in the immediate ART strategy [10]. The WHO guidelines were subsequently revised in 2008 to recommend immediate ART in all HIV-infected infants under 12-months, irrespective of clinical or immune status [11]. In 2010, this was extended to all HIV-infected children under 2-years [12] and in 2013, to all under 5-years [13].

Early initiation of ART during infancy, when risk of mortality is highest, requires access to early infant HIV diagnosis (EID) based on virologic assays (e.g. DNA PCR or RNA assays) rather than standard serology tests due to persistence of maternal anti-HIV antibodies for up to 18-months [11]. This requires access to a specialised laboratory and trained technicians. In 2011, it was estimated that only 28% of HIV-exposed infants in resource limited countries received EID within the first two months of life, as per recommendations [14], and coverage of ART among children eligible for treatment remains disproportionately low at 28% as compared to 57% among adults [1].

As part of the UN Global plan for the virtual elimination of paediatric AIDS through the scale up of prevention of MTCT (PMTCT) services, there is an urgent need to improve access to EID and ensure timely provision of ART in HIV-infected children. To date there are no data on the cost-effectiveness of early HIV diagnosis and treatment strategies in children in resource-limited settings to inform donors and policy makers facing competing public health demands.

This study examines the cost-effectiveness of EID of HIV-exposed infants and immediate ART of HIV-infected children under 24-months in the Thai setting. Thailand was one of the first middle-income countries to pilot a national EID programme from 2007 [15].

## Methods

We examined the cost-effectiveness, from the health care provider’s perspective, of: (i) EID of HIV-exposed infants using DNA PCR and immediate ART in HIV infected children <24-months (Early-Early); (ii) EID and deferred ART based on clinical and immune criteria (Early-Late); as compared to (iii) clinical based diagnosis and serology at 18-months with deferred initiation of ART based on clinical and immune criteria (Reference). The reference strategy represented standard of care in Thailand up to 2007 and reflects the current status in many resource-limited settings without access to EID. The Early-Late strategy represented the intermediate stage where EID is provided but with deferred ART as per 2008 WHO guidelines, before the results of the CHER trial [16]. This reflects current practice in some settings expanding EID, where immediate ART is not implemented due to poor referral systems or lack of readiness of parents/caregivers [17]. The Early-Early strategy reflects the 2010 WHO recommendations for best practice [12]. This is similar to current Thai guidelines which recommend EID and immediate ART in HIV infected children <12-months irrespective of immune/clinical status, although this has not yet been extended to all children <24 months [18].

### Model Overview

**Figure 1:**
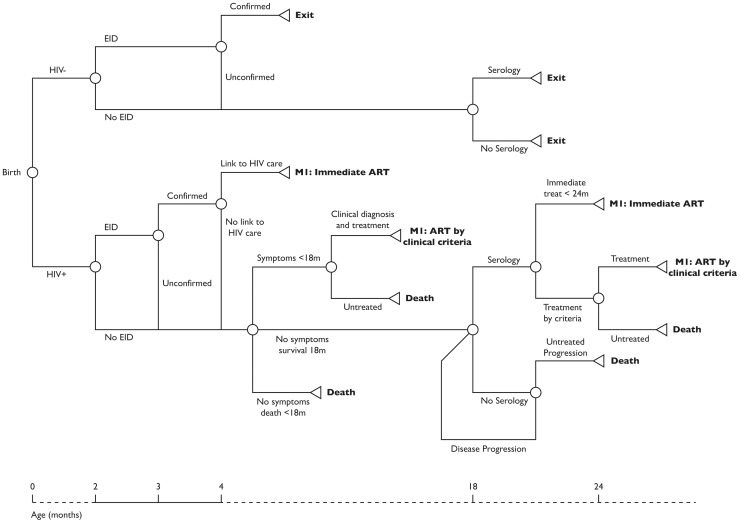
Decision tree for HIV diagnosis and treatment strategies.

**Figure 2:**
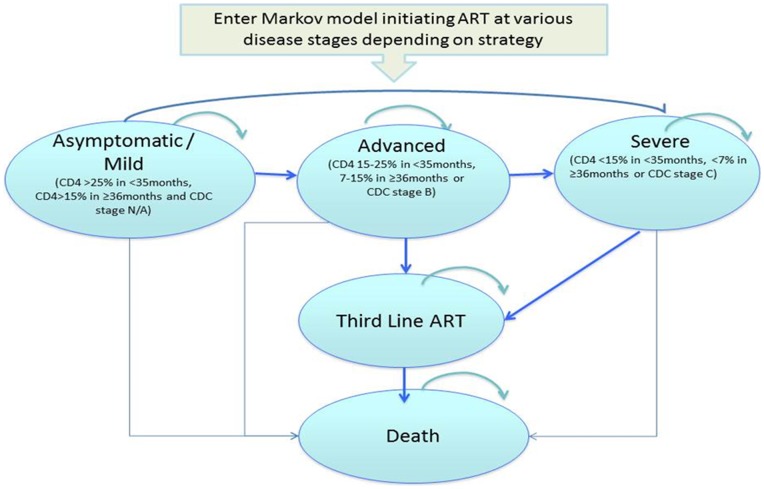
Markov model for HIV diagnosis and treatment strategies.

We developed a cohort simulation model that incorporates data on perinatal transmission, natural history of HIV disease, treatment efficacy and cost of care from Thailand. The model was composed of a decision tree and a Markov model. The decision tree (Figure 1) presents the diagnostic component of the intervention and includes all HIV-exposed infants. The Markov model (Figure 2) presents the ART component of the intervention and includes only HIV-infected children initiated on therapy. Based on the current estimates in Thailand, we assumed a hypothetical cohort of 6,000 children born from HIV-infected mothers per year [14].

### Decision Tree

HIV-exposed children entered the decision tree at birth with a probability of HIV infection through in-utero or intra-partum transmission. Post-partum transmission through breastfeeding was not considered as Thailand has very high coverage of formula feeding for this population.

Early HIV diagnosis was provided using DNA PCR on dried blood spots (DBS) with an assumed 100% sensitivity and specificity [19]. In the Early-Early and Early-Late strategies HIV-exposed children had probabilities of routine EID at 6–8 weeks of age, with confirmation EID as soon as possible in children who test positive (within one month). Among children who tested negative, the second confirmation test was conducted at 4 months [18]. HIV-infected children with confirmed diagnosis had monthly probabilities of: linkage to HIV care and initiation of ART (as per criteria in each strategy) or pre-ART death.

In the reference strategy, HIV-infected children had monthly probabilities of developing HIV symptoms; clinical diagnosis if <18-months and serology thereafter; routine serology test at 18-months; initiation of ART based on clinical criteria or pre-ART death. Due to incomplete coverage of EID and linkage to HIV care, a proportion of HIV-infected children in the early diagnosis strategies would revert to the reference strategy with probabilities of disease progression and clinical based diagnosis but with access to EID for confirmation of HIV-infection if <18-months. The analytical time horizon for costs and life years among HIV-infected children in the absence of ART ran from birth until all children died or started therapy.

In all strategies, HIV-uninfected children were assumed to have the same probability of routine HIV diagnosis and exit the model at time of diagnosis or at 18-months if undiagnosed. They contributed only to the cost of EID and incurred no mortality as we assumed their survival to be unaffected by the different strategies.

### Markov Model

HIV-infected children diagnosed and initiating ART entered the Markov model in one of the following three live states which represents their disease status at start of therapy:

- Asymptomatic/Mild: CD4>25% if <35 months or CD4>15% if ≥36 months or Centre of Disease Control (CDC) clinical stage N or A
- Advanced: CD4 15–25% if <35 months, 7–15% if ≥36 months or CDC clinical stage B
- Severe: CD4<15% if <35 months, <7% if ≥36 months or CDC clinical stage C

The model was based on monthly cycles with a probability of remaining in the same health state, advancing to more severe health state or death. We assumed non reversibility of health states as children starting ART at more advanced disease stage are at higher risk of mortality during the first year of therapy [4], [6], [9], have lower probability of long-term immune reconstitution and experience longer duration in immunocompromised state despite ART [20], [21].

The probability of death on ART by health state was based on the PHPT cohort (NCT00433030) with a median 5-years of follow up on therapy (described below) [9], and extrapolated using a Weibull distribution. The model projected unrealistically high long-term survival on therapy, most likely due to lack of treatment failures and long-term mortality captured during the follow up time. To allow for this, we assumed that after five years of ART, children in the advanced and severe disease stage had a probability of failing their first and second line therapy and progressing to the ‘Third line ART’ state, with a higher risk of mortality to reflect the increased risk of sub-optimal adherence and viremia over time on therapy. Children in the asymptomatic/mild stage were assumed to experience disease progression before progressing to Third line ART. The Markov model ran for up to 40 years on ART.

### Population

As much as possible the modelling was based on data from Thailand, primarily from the PHPT paediatric observational cohort study, which has been described elsewhere [9], [22]. In brief, HIV-infected children were enrolled through two modes of entry. First was the Birth cohort, children born to HIV-infected mothers enrolled in clinical trials on PMTCT [23], [24] received EID at birth and at 6 weeks, HIV-infected children initiated ART based on WHO 2006 immune/clinical criteria [16]. Second was the Referred cohort: children without access to EID, who were diagnosed after presentation with HIV symptoms or through routine serology testing ≥18 months, they also initiated ART based on immune/clinical criteria. Due to limited data on long-term survival based on the Early-Early diagnosis and immediate ART strategy as part of routine care, this was modelled based on data from the CHER trial [10].

### Input Parameters

Key input parameters are shown in Table 1. Coverage of EID using DNA PCR on DBS were based on data from the Thai national EID programme [15], [25].

**Table 1:** Input parameters.

	Estimate	Distribution	Source
**Perinatal HIV transmission and coverage of HIV diagnosis**			
Rate of mother to child transmissionof HIV in Thailand	3.9% (95% CI, 2.2–6.6)	Beta	[36].
Coverage of early infant HIV diagnosis	68% (range 47–79).	Normal	[15], Range [14]
Confirmation of EID	78% (range 47–85)	Normal	[15], Range [17]
Linkage to HIV care within 3 months ofearly diagnosis	73.1% (95% CI,64–82)	Beta	[25]
Initiated ART within 3 months of linkage to HIV care	85.4% (range 79–92)	Beta	[25]
Coverage of clinical diagnosis <18 months amongsymptomatic	80% (range 70–90)		Assumption
Coverage of serology testing >18 months amongsymptomatic	95% (range 90–97)		Assumption
Coverage of routine serology testing at 18 months	75.8% (range 70–80)		[36], Range assumption
**Probability of developing symptoms when untreated (monthly)**			
Probability of developing symptoms <12-monthswhen untreated	6.4% (95% CI, 5.5–7.2)	Beta	[29]
Probability of developing symptoms between12–23 months when untreated	3.2% (range, 2.8–3.6)		Assumption based on half rate of <12 months.
**Distribution of disease stage at start of ART by strategy**			
Reference strategy: Under 12 months; Over12 months	A: 8%; 8%; B: 31%; 24%;C: 62%; 67%	Dirichlet	PHPT Referred cohort
Early-late strategy: Under 12 months; Over12 months	A: 28%; 26%; B: 43%; 40%;C: 28%; 34%	Dirichlet	PHPT Birth cohort
Early-early strategy: Under 12 months; Over12 months	A: 66%, 26%; B: 27%, 40%;C: 7%, 34%	Dirichlet	Assumption: based on PHPT Birthcohort* CHER study risk ratio 0.25in <12 months [10]
**Monthly probability of disease progression on ART**			
Stage A to B	0.43%		[61]
Stage A to C	0.08%		
Stage B to C	0.14%		
Stage B or C to third line after 5 yearsof ART	0.83%		PHPT cohort
Third line to death			[62]
Risk reduction in disease progression	0.25 (95% CI, 0.15–0.41)		[10]

*Note: EID; early infant HIV diagnosis, ART; antiretroviral therapy, CDC; centre of disease control*.

### Survival Estimates and Disease Progression

Survival among untreated HIV-infected children was based on natural history data from the Birth cohort with children censored at date of death, last seen alive or start of ART, whichever was earliest (Table S1 in File S1). Due to few children alive and untreated after 2-years of age, the survival estimates after 2-years were based on adult natural history survival [26].

Survival estimates in HIV-infected children receiving ART were based on the Birth cohort for the Early-Late strategy and the Referred cohort for the Reference strategy. In both strategies, risk of death was highest during the first year of therapy and declined to low levels thereafter. Risk of mortality was substantially higher in children initiated on ART based on clinical/immune criteria <12-months old (rapid progressors) as compared to older children who survived infancy without ART (slow progressors). To reflect this we weighted the experience of two subgroups (under and over 12-months at start of ART) to create a base case (Table S2 in File S1). In the Early-Early strategy, where infants receive immediate ART upon diagnosis, it is unknown what proportion of children would have been rapid or slow progressors, and this is likely to vary across settings according to different distributions of in-utero and intra-partum transmission [26]–[29]. We assumed a 50∶50 distribution and in sensitivity analysis we tested different distribution assumptions. In the CHER trial, the 76% risk reduction in mortality observed in the immediate treatment strategy was driven by the reduction in pre-ART death (among untreated children) which is captured in the decision tree. There was no evidence of a difference in mortality after the start of therapy [10], therefore no reduced risk of mortality on ART was applied to the early-early strategy of the Markov model. However, a risk reduction on disease progression was applied for the first 12 months of therapy to reflect the results of the CHER study’s 40 weeks follow up after the start of immediate ART.

The distribution of disease stage at start of therapy in the Reference and Early-Late strategies was based on that observed in the PHPT cohort (Table 1). The distribution in the Early-Early strategy was based on the Birth cohort with the risk reduction in disease progression of 0.24 observed in the CHER study applied to children in the Advanced and Severe stages starting ART <12-months-old. Although CDC stage B and C and CD4<25% were exclusion criteria in the CHER study, we wanted to allow for the natural disease progression in the pre-ART period to occur to avoid over-estimating the benefits of the intervention. Indeed, in the CHER trial, 22.5% of children were excluded from randomization (<12 weeks old) due to advanced disease at screening (2.9% CDC stage C, 19.6% had CD4<25%), who may still benefit from early treatment in routine care and therefore were included in the model.

### Cost Parameters

The cost estimates used in this model are listed in Table 2. All costs were adjusted for inflation for Thailand up to 2011 and converted to US dollars using purchasing power parity (17.5 baht per international US dollar) [30]. As much as possible costs were based on data from the PHPT cohort (EID, hospitalization and ARV drug costs). The unit cost of EID was based on DNA PCR in-house assays [19], estimated at $57.14 (1000 baht) per test including cost of initial investment in equipment, reagents, DBS, transportation costs (DBS transported by regular postal mail), human resources and maintenance of equipment [25]. Standard serology test was estimated at $1 per test [31]. Mean cost of antiretroviral drugs (first, second and third line regimens) was based on annual average cost observed [19]. Cost of hospitalization of children on ART varied according to disease stage during the first year of therapy, and a mean cost thereafter [22]. The cost of pre-ART death was assumed to be equal to the cost of hospitalization during the first year of therapy of a child in the severe disease stage, as observed in adult studies [32]. We did not apply a cost of death on ART as we assumed this to be already incorporated in the hospitalization cost estimates.

**Table 2:** Cost parameters.

Costs (2011 US$)	Unit cost	Source
**Pre and post HIV test counselling**		
HIV positive result	$9.53 (range 5.02–19.12)	[38]
HIV negative result	$3.61 (range 2.06–10.24)	
**Cost of HIV diagnosis per test**		
Early infant HIV diagnosis using DNA PCR and dried blood spots	$57.14	[25]
HIV rapid test by serology	$1	[31]
**Mean cost of antiretroviral drugs per child per month**		
Mean cost during the first five years of therapy (includes first and second line therapy)	$61.10 (SE 61.10)	[63]
Mean cost after five years of therapy (includes first and second line therapy)	$86.30 (SE 86.30)	[63]
Third line ART	$148.30 (SE 148.30)	[63]
Laboratory monitoring on ART	$26.09 (SE 26.09)	[52] and PHPT unpublished data.
Hospitalization during first year of ART in disease stage A or B	$24.9 (SE 24.90)	[22]
Hospitalization during first year of ART in CDC stage C	$43.0 (SE 43.0)	[22]
Hospitalization after first year of ART (all disease states)	$5.20 (SE 5.20)	[22]

Note. All cost estimates were adjusted for inflation up to 2011.

### Model Validation, Cost-effectiveness and Sensitivity Analyses

Model validation was based on model projections of survival at 1 and 5 years of ART as compared to the PHPT cohort data for the Early-Late and Reference strategies.

The modelled costs and outcome, in terms of life years gained were discounted at 3% per year [33]. We report the projected discounted and undiscounted life expectancy per HIV infected child, discounted total programme costs and lifetime costs per HIV infected child were compared across the three strategies. The incremental cost-effectiveness ratio (ICER) was defined as difference in discounted total programme cost divided by difference in discounted total life years gained (LYG). An ICER of less than one times the Gross Domestic Product (GDP) per capita for Thailand (US $4,420 in 2011, [30]) was considered as cost effective [34].

We conducted deterministic univariate sensitivity analysis using the high and low estimates of key input parameters to assess the impact on the cost-effectiveness estimates. Best and worse-case scenarios were assessed using high and low estimates of perinatal transmission combined with current and high estimated costs of EID and ART.

Probabilistic sensitivity analysis taking into account uncertainty of all input parameters was conducted using a Monte Carlo simulation with 1,000 random draws from the specified parameter distribution. Cholesky decomposition of the variance-covariance matrices was used to capture correlation between coefficients in the regression model for mortality on ART [35]. The results are presented in cost-effectiveness acceptability curves which represent the probability of the interventions being cost effective at various willingness to pay thresholds. In addition, sub-group analyses were conducted to assess the cost-effectiveness of the interventions by varying levels of access to PMTCT services and risk of perinatal transmission.

## Results

### Model Validation

Projected survival at 1 and 5 years of ART in the Reference and Early-Late strategy were compared to the survival estimates in the PHPT cohort according to age at start of therapy. Among children initiated on ART under 12-months old, the model projected poorer survival as compared to that observed in PHPT cohort, but projections were within the 95% confidence interval of the survival estimate, most likely due to the small sample size in this age group(Table S3 in File S1). Among children initiated on ART after 12-months of age, projected survival was within 2% of that observed in the PHPT cohort.

### Projected Life Expectancy and Cost-effectiveness

In the reference strategy, the discounted life expectancy of an HIV-infected child was 13.3 years (undiscounted, 21.0 years), with a discounted lifetime cost of $17,335 per child. In the Early-Late strategy, the life expectancy increased to 14.3 years (undiscounted 22.8 years), with a lifetime cost of $22,583 per child. In the Early-Early strategy, the life expectancy increased further to 17.8 years (undiscounted 29.1 years), with a lifetime cost of $29,108.

The Early-Late strategy had an incremental cost-effectiveness ratio of $5,149 per LYG as compared to the Reference strategy (Table 3). The Early-Early strategy had an ICER of $2,615 per LYG compared to the Reference strategy and $1,873 per LYG compared to the Early-Late strategy. The Early-Late strategy was extendedly dominated as compared to Early-Early strategy and therefore was not considered further (Figure S1 in File S1).

**Table 3:** Cost and cost-effectiveness of the intervention strategies.

Programme model	Reference	Early-Late	Early-Early
Cost of HIV Diagnosis & pre-ART death	$23,754	$454,010	$458,433
Cost of ART including hospitalization	$4,009,804	$4,800,673	$6,314,682
Total Cost (All children)	$4,033,558	$5,254,683	$6,773,115
Total LY (HIV+child)	3,086	3,323	4,134
Incremental cost-effectiveness ratio per LY over Reference	–	$5,149	$2,615
Incremental cost-effectiveness ratio per LY over Early-Late	–	–	$1,873

Note: Model assumes 6,000 children born to HIV infected mothers with a risk of HIV transmission of 3.9% and provision of lifelong ART among HIV infected children diagnosed and initiated on therapy. All costs converted to USD using purchasing power parity (17.5 baht per international US dollar).

Based on the assumption of 6,000 HIV infected pregnant women delivering in Thailand per year and an overall risk of mother to child transmission of HIV of 3.9%, the total discounted programme cost was estimated at $4.0 million in the Reference strategy and increases to $6.8 million in the Early-Early strategy (Table 3). However, over 90% of the total cost of the Early-Early strategy was attributed to lifetime cost of ART for HIV infected children and less than 10% on the early infant HIV diagnosis component for all HIV-exposed infants.

### Sensitivity Analyses

In univariate sensitivity analysis, the ICER of the Early-Early strategy was most sensitive to the discount rate, the cost of antiretroviral drugs, laboratory monitoring, cost of EID and rate of perinatal HIV transmission (Figure S2 in File S1). However, under all scenarios the ICER remained under $4,500 per LYG. These results were supported by the probabilistic sensitivity analysis allowing for uncertainty of all model parameters, the cost-effectiveness acceptability curve show a 99% probability of the Early-Early strategy being cost effective at $4,500 per LYG (Figure S3 in File S1).

**Figure 3:**
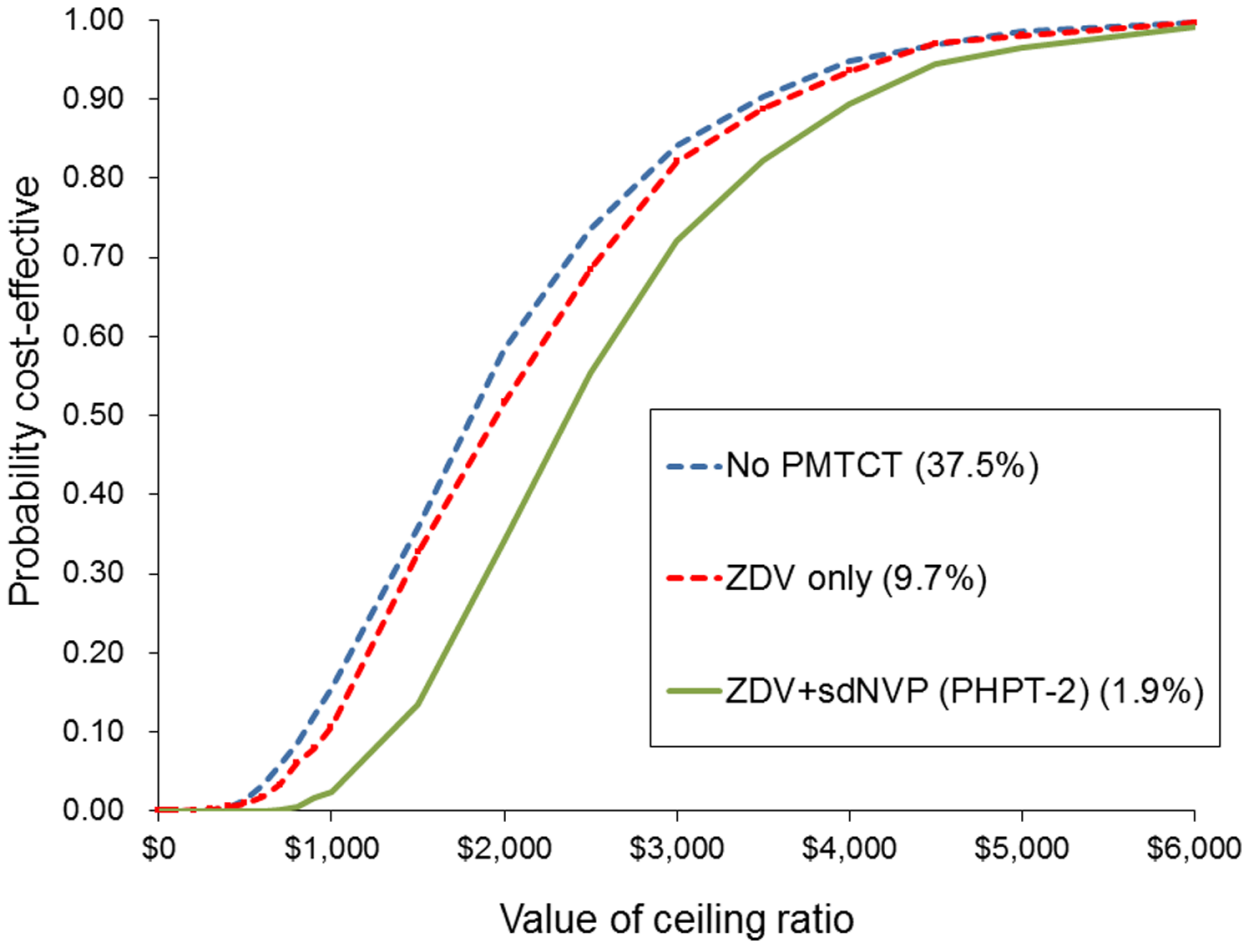
Cost-effectiveness acceptability curve of Early-Early versus Reference strategy by PMTCT prophylaxes and risk of perinatal transmission.

In sub-group analyses, the cost-effectiveness of the Early-Early strategy was assessed according to varying levels of coverage of prophylaxis for PMTCT and risk of perinatal transmission as observed in Thailand [36]. The Early-Early strategy was most cost effective among children at highest risk of HIV infection, i.e. those who received no PMTCT, with 37.5% risk of perinatal transmission. The ICER of the Early-Early intervention in this population was $2,248 per LYG as compared to the Reference strategy. With improved prophylaxis for PMTCT and reduced risk of perinatal transmission, the ICER increased slightly, but the Early-Early strategy remained cost effective as compared to the Reference strategy at under $4,500 per LYG across all sub-groups (Figure 3).

In multi-way sensitivity analysis, we assumed the overall perinatal HIV transmission rate reduced to a target rate of 1.5% with the introduction of universal HAART for PMTCT [37], high estimates of EID coverage, confirmation and linkage to ART and current cost of EID and ART. In this scenario, the ICER increased to $3,470 per LYG, while the total programme cost reduced to $3.0 million due to fewer HIV-infected children requiring lifetime ART.

## Discussion

In this study we modelled the survival and costs of providing EID using DNA PCR and immediate or deferred initiation of ART in HIV infected children aged <24-months as compared to late HIV diagnosis based on clinical status or serology at 18-months and deferred initiation of ART based on clinical and immune criteria in the Thai setting. The EID and immediate ART strategy increased the discounted life expectancy of HIV infected children from 13.3 to 17.8 years, at an incremental cost-effectiveness ratio of $2,615 per LYG in the base case. This is approximately half of Thailand’s GDP per capita and would be considered as cost effective under WHO recommendations [34].

Importantly, these estimates were made by converting all costs to US dollars using purchasing power parity (17.5 baht per international USD). If we had used market exchange rates (34.3 baht per USD) as in other studies [38], with the rationale that the majority of costs are attributed to imported antiretrovirals and laboratory assays that are subject to market exchange rates, then the Early-Early strategy would have even lower incremental cost-effectiveness ratio and lower programme costs (Table S4 in File S1).

The benefits of the Early-Early strategy were observed in two main areas. Firstly, EID and immediate ART minimized the period in which HIV-infected children were untreated during infancy, resulting in a halving of pre-ART deaths from 42% in the Reference strategy down to 21% in the Early-Early strategy. This figure is not lower due to existing gaps in EID coverage and referral of HIV infected infants for ART initiation (data not shown). Second was the reduction in early mortality on ART due to fewer children initiating therapy at advanced disease stage. The cost-effectiveness estimate of this strategy is likely to be under-estimated as we assumed the benefit of immediate ART in reducing disease progression would only persist for the first year of therapy, based on the follow up duration of the CHER trial. It is likely that the benefits are longer lasting due to the preservation of the immune system; children are better able to maintain good long-term immunologic response to ART as reported in observational cohorts of infants who initiated therapy during the first 3 months of life while asymptomatic in Europe and the US [39], [40]. Furthermore, we have not taken into account the benefits of averting damage to cognitive function and neurological development among children who progress to advanced disease when left untreated[41]–[44], nor have we included the benefits in terms of quality of life, of accessing early infant HIV diagnosis among HIV-uninfected infants.

The Early-Late strategy where EID was provided but ART deferred till after meeting clinical and immune criteria, as conducted in the PHPT birth cohort prior to WHO 2008 guidelines, was less cost effective when compared to the Reference Strategy. It resulted in a limited increase in the discounted life expectancy of HIV infected children (from 13.3 years in the reference strategy to 14.3 years). This is most likely due to the limited impact on reducing pre-ART deaths among infants who have high risk of rapid disease progression and death even at high CD4% [45], with no prior signs and symptoms [10], [29]. In addition, infants who initiate therapy after disease progression remain at higher risk of mortality despite ART [7]–[9]. This highlights the importance of effective referral of HIV-infected infants as soon as they are diagnosed for immediate ART to maximize the potential benefits of early treatment.

To our knowledge, this is the first cost-effectiveness evaluation of early infant HIV diagnosis and different treatment strategies in children. Previous studies have examined the cost and acceptability of early HIV diagnosis using DNA PCR in low- and middle- income countries [17], [46], [47]. One study examined the cost-effectiveness of early infant HIV diagnosis using rapid antibody tests and clinical examination, primarily to screen out HIV-uninfected children at minimal cost, however that strategy had poor specificity among infants under 6 months and only assessed the cost-effectiveness per correct diagnosis and did not consider provision of ART for infected children [48]. An economic sub-study in the CHER trial reported that cost of earlier provision of ART to asymptomatic infants was more than offset by the reduced cost of inpatient care as compared to the deferred ART strategy, but did not include the cost of early HIV diagnosis of all exposed infants [49].

While there is a growing body of literature on the cost-effectiveness of different treatment strategies in HIV-infected adults [50], there are only two comparable studies on HIV care in children. One was on the cost-effectiveness of cotrimoxazole prophylaxis for prevention of opportunistic infections in untreated HIV infected children in Zambia ($74 per LYG) [51]. The second was on cost-effectiveness of virological monitoring and provision of second line therapy in HIV infected children in Thailand ($3,393 per year of virological failure averted) [52]. The latter is not directly comparable to our study as we assumed that HIV infected children on ART received routine CD4 and virological monitoring every 6 months and had access to second and third line regimen upon treatment failure as per national guidelines [18]. Based on these assumptions, the addition of EID and immediate treatment in all HIV-infected children <24-months was cost-effective, and is likely to be affordable in the Thai setting. Importantly, if the rate of mother-to-child transmission continues to decline further with introduction of HAART for PMTCT and more extensive provision of HAART to HIV-infected adults (including women during time of conception and pregnancy) [53], then the total programme cost of this strategy is likely to decrease over time as fewer children are infected and require lifelong treatment which accounts for over 90% of the programme costs.

In addition, on-going developments in low cost HIV laboratory services including EID at point of care in resource-limited settings are likely to further simplify collection and transportation of samples and make EID more affordable and feasible for routine use [54]. Maturing EID programmes have reported innovative strategies to improve uptake of EID and retention of HIV-exposed infants, although there remain scarce data on the follow-up and treatment status of newly diagnosed HIV-infected infants [15], [55], [56]. Such indicators are critical for the evaluation of PMTCT and paediatric HIV programmes and should be highlighted as an important goal as part of the campaign for a zero HIV generation.

There are a number of important limitations of this study. First, the survival estimates of children on ART were extrapolated from an observational study with five years of follow-up due to scarce long-term data from a low and middle income setting. Data from paediatric cohorts in the US or Europe were not used as they represented a different population with access to earlier treatment using more potent and costly drugs and lower estimates of mortality and hospitalization [5], [57]. Second, the Markov model of children on ART assumed non reversibility of health states, as there remains limited data on long-term immunologic response and risk of mortality among infants/older children starting therapy at different disease stages to inform a more complex reversible model. However, the main benefits of EID and immediate ART were the reduction in pre-ART deaths and early mortality on ART, therefore a more detailed model of the long-term survival is unlikely to affect the overall findings.

Third, this study is based on data largely from the Thai setting and therefore the findings cannot be extended to other settings with different coverage of services, mortality rates, costs and thresholds for cost-effectiveness. Furthermore, this study was based on a non-breastfeeding population, most of the countries with the highest burden of HIV in sub-Saharan Africa, recommend exclusive breastfeeding and thus repeated early infant HIV diagnosis during the breastfeeding period and after weaning would be required. Fourth, in this study we assumed 100% sensitivity and specificity of DNA PCR testing from 2 months of life – based on data from the PHPT study where children were exposed to nevirapine and zidovudine prophylaxis for PMTCT. A study by Shapiro and colleagues suggests that early diagnosis of infants exposed to maternal or infant HAART for PMTCT may be less sensitive during the first months of life [58], which may have important implications for the recommended schedule for early diagnosis and may require more confirmation tests. However, when we assumed a doubling in the cost of EID – this intervention was still cost effective. Lastly, recent reports of a functional cure of an HIV-infected infant diagnosed and initiated ART at 30 hours of life in the United States [59] has generated much interest in the potential benefits of birth testing and very early ART in preventing seeding of the HIV reservoir [60]. However, the sensitivity of virological tests at birth with DBS and exposure to maternal HAART are not well described. Also, the birth test would only identify the in-utero transmissions, and the feasibility of such rapid return of test results and ART referral in resource-limited settings has yet to be determined; implementation studies and cost-effectiveness analyses of a birth test algorithm are needed to inform future policies and programmes.

## Conclusion

Early infant HIV diagnosis combined with immediate ART of children under 24 months was cost effective in the Thai setting as compared to late diagnosis and deferred treatment. Expanding programmes for EID must place greater emphasis on retention of HIV infected infants identified and timely initiation of ART prior to disease progression to maximize the benefit in reducing HIV related morbidity and mortality in this highly vulnerable population.

## Supporting Information

File S1 Contains Table S1, Table S2, Table S3, Table S4, Figure S1, Figure S2, Figure S3.
(DOCX)
