# Supplementary material for: Cost-Effectiveness of Early Infant HIV Diagnosis of HIV-Exposed Infants and Immediate Antiretroviral Therapy in HIV-Infected Children under 24 Months in Thailand
Source: PLoS One. 2014 Mar 14;9(3):e91004. doi: 10.1371/journal.pone.0091004 (PMC3954590; doi:10.1371/journal.pone.0091004)
Supplement: File S1 — Contains Table S1, Table S2, Table S3, Table S4, Figure S1, Figure S2, Figure S3. (DOCX) [file pone.0091004.s001.docx]

**Supplementary Tables and Figures**

**Table S1. Regression models**

| **Weibull models** | **Estimate (SE)** | **Source** |
| --- | --- | --- |
| **Monthly probability of pre-ART death** |  | PHPT Birth cohort pre-ART |
| Constant | -3.36 (0.36) |  |
| Birth weight | -0.133 (0.03) |  |
| Lambda | 0.04 |  |
| Gamma | 0.66 (0.11) |  |
| **Monthly probability of death on ART** |  | PHPT cohort on ART |
| Constant | -3.99 (-3.65) |  |
| Age <12-months at start of ART* | 2.91 (2.23) |  |
| CD4% over 7% at start of ART | -0.13 (-0.13) |  |
| Weight to height z-score over -0.4 at start of ART (only if age >12 months) | -0.77 (-0.71) |  |
| Lamda stage A <12months | 0.02 |  |
| Lamda stage B <12months | 0.08 |  |
| Lambda stage C <12months | 0.11 |  |
| Lambda stage A ≥12 months | 0.003 |  |
| Lambda stage B ≥12 months | 0.01 |  |
| Lambda stage C ≥12 months | 0.02 |  |
| Gamma |  |  |

*Note. ART; antiretroviral therapy, SE; standard error. *Effect of <12months applied for first 3 years of therapy in Reference and Early-Late strategy and among rapid progressors in Early-Early strategy.*

**Table S2. Base Case characteristics by age and disease stage at start of therapy**

|  | **Age at start of ART** | | |
| --- | --- | --- | --- |
|  | **Infant (<12 months)** | **Non infant (7 years)** | |
| **Stage Asymptomatic/Mild** |  | |  |
| CD4% | 28% | | 20% |
| Weight-for-height z-score | - | | 0 |
| **Stage Advanced** |  | |  |
| CD4% | 18% | | 15% |
| Weight-for-height z-score | - | | -0.2 |
| **Stage Advanced** |  | |  |
| CD4% | 16% | | 7% |
| Weight-for-height z-score | - | | -0.5 |

*Note: ART; antiretroviral therapy. Weight for height z-score at start of ART was not associated with mortality among infants but was strongly associated in older children.*

**Table S3. Model validation of survival at 1 and 5 years after start of ART by strategy and age group.**

|  | PHPT cohort survival (95%CI) | | Model projected survival | |
| --- | --- | --- | --- | --- |
|  | 1 year | 5 years | 1 year | 5 years |
| **Age<12 mo. at start ART** |  |  |  |  |
| Reference | 84.6 (51.2-95.9) (n=12) | 84.6 (51.2-95.9) (n=5) | 78% | 67% |
| Early-Late | 84.1 (69.5-92.1) (n=41) | 74.1 (58.0-84.8) (n=25) | 82% | 73.6% |
| Early-Early | - | - | 93.6% | 90.0% |
| **Age≥12-mo. at start ART** |  |  |  |  |
| Reference | 95.3 (92.8-96.9) (n=415) | 93.6 (90.7-95.6) (n=178) | 96.0% | 92.5% |
| Early-Late | 98.5 (89.6-99.8) (n=62) | 96.7 (87.4-99.2) (n=39) | 97.4% | 95.0% |
| Early-Early | - | - | 97.4% | 95.0% |

Note: ART; antiretroviral therapy

**Table S4. Costs and cost-effectiveness of the intervention strategies when using market exchange rates for cost estimates.**

| Programme model | Reference | Early-Late | Early-Early |
| --- | --- | --- | --- |
| Cost of HIV Diagnosis & pre-ART death | $22,693 | $273,014 | $275,605 |
| Cost of ART including hospitalization | $2,403,203 | $2,847,480 | $3,709,853 |
| Total Cost (All children) | $2,425,896 | $3,120,493 | $3,985,458 |
| Total LY (HIV+ child) | 3,086 | 3,323 | 4,134 |
| Incremental cost-effectiveness ratio per LY over Reference | - | $2,929 | $1,489 |
| Incremental cost-effectiveness ratio per LY over Early-Late | - | - | $1,067 |

*Note: LY; life year, ART; antiretroviral therapy. Model assumes 6,000 children born to HIV infected mothers with a risk of HIV transmission of 3.9% and provision of lifelong ART among HIV infected children diagnosed and initiated on therapy. Market exchange rate (34.3 baht per US dollar) applied for all costs.*

**Figure S1. Probabilistic sensitivity analysis: Monte Carlo simulation of incremental life years gained and incremental cost as compared of Early-Late and Early-Early versus Reference strategy.**

**Figure S2. Univariate Sensitivity Analysis: Effect of input parameter high and low estimate on ICER in Early-Early versus Reference arm**

**Figure S3. Cost-effectiveness acceptability curve**
